# Supplementary material for: High Levels of Antibiotic Resistance in MDR-Strong Biofilm-Forming Salmonella Typhimurium ST34 in Southern China
Source: Microorganisms. 2023 Aug 3;11(8):2005. doi: 10.3390/microorganisms11082005 (PMC10458675; doi:10.3390/microorganisms11082005)
Supplement: Supplementary file 1 [file microorganisms-11-02005-s001.zip › microorganisms-2483820-supplementary.pdf]

Table S1. Biofilm formation of the 295 *S.* Typhimurium isolates.

Biofilm Formation	Source (%)		Total Number of Isolates
	Food	Human	
None	6 (4.58%)	16 (9.76%)	22 (7.46%)
Weak	33 (25.19%)	19 (11.59%)	
Moderate	10 (7.63%)	33 (20.12%)	273 (92.54%)
Strong	82 (62.60%)	96 (58.54%)	
Total number	131	164	295

Table S2. Antimicrobial resistance in *S.* Typhimurium isolates from patients.

Regions	Percentage (%) of Resistant Isolates														
	AMP	FEP	CTX	CN	AK	STR	NAL	CIP	OFX	FFC	C	PB	SUL	TET	IPM
Shanghai (n = 88)	76.14%	1.14%	9.09%	27.27%	0.00%	44.32%	55.68%	15.91%	7.95%	55.68%	51.14%	3.41%	82.95%	53.41%	0.00%
Chongqing (n = 45)	95.56%	0.00%	37.78%	26.67%	0.00%	48.89%	62.22%	33.33%	17.78%	68.89%	68.89%	4.44%	93.33%	60.00%	0.00%
Guangxi (n = 29)	82.76%	0.00%	17.24%	51.72%	0.00%	31.03%	100.00%	31.03%	27.59%	100.00%	65.52%	3.45%	89.66%	100.00%	0.00%
Total (n = 162)	82.72%	0.62%	18.52%	31.48%	0.00%	43.21%	65.43%	23.46%	14.20%	67.28%	58.64%	3.70%	87.04%	63.58%	0.00%

Table S3. Antimicrobial resistance in *S.* Typhimurium isolates from Food.

Regions	Percentage (%) of Resistant Isolates														
	AMP	FEP	CTX	CN	AK	STR	NAL	CIP	OFX	FFC	C	PB	SUL	TET	IPM
Guangdong (n = 72)	27.78%	2.78%	2.78%	31.94%	0.00%	56.94%	51.39%	13.89%	23.61%	66.67%	51.39%	4.17%	81.94%	100.00%	0.00%
Shanghai (n = 36)	61.11%	2.78%	5.56%	25.00%	0.00%	33.33%	75.00%	13.89%	16.67%	52.78%	44.44%	0.00%	77.78%	72.22%	2.78%
Sichuan (n = 25)	96.00%	0.00%	4.00%	4.00%	0.00%	92.00%	100.00%	8.00%	84.00%	100.00%	4.00%	0.00%	100.00%	100.00%	0.00%
Total (n = 133)	49.62%	2.26%	3.76%	24.81%	0.00%	57.14%	66.92%	12.78%	33.08%	69.17%	40.60%	2.26%	84.21%	92.48%	0.75%

Table S4. Antimicrobial resistance of Different STs of *S.* Typhimurium.

STs	AMP	FEP	CTX	GEN	AK	NAL	CIP	OFX	FFC	C	STR	PB	SUL	TET	IPM
ST19	15 (31.91%)	1 (2.13%)	1 (2.13%)	4 (8.51%)	0 (0.00%)	15 (31.91%)	0 (0.00%)	1 (2.13%)	20 (42.55%)	19 (40.43%)	10 (21.28%)	0 (0.00%)	30 (63.83%)	17 (36.17%)	0 (0.00%)
ST34	184 (75.31%)	3 (1.23%)	32 (13.17%)	80 (32.92%)	0 (0.00%)	179 (73.66%)	55 (22.63%)	66 (27.16%)	184 (75.72%)	130 (53.50%)	136 (55.97%)	9 (3.70%)	223 (91.77%)	209 (86.01%)	1 (0.41%)
ST36	2 (100.00%)	0.00%	2 (100.00%)	0.00%	0 (0.00%)	0 (0.00%)	0 (0.00%)	0 (0.00%)	0 (0.00%)	0 (0.00%)	0 (0.00%)	0 (0.00%)	0 (0.00%)	0 (0.00%)	0 (0.00%)
ST99	0 (0.00%)	0 (0.00%)	0 (0.00%)	0 (0.00%)	0 (0.00%)	0 (0.00%)	0 (0.00%)	0 (0.00%)	0 (0.00%)	0 (0.00%)	0 (0.00%)	0 (0.00%)	0 (0.00%)	0 (0.00%)	0 (0.00%)
ST1557	0 (0.00%)	0 (0.00%)	0 (0.00%)	0 (0.00%)	0 (0.00%)	2 (100.00%)	0 (0.00%)	0 (0.00%)	0 (0.00%)	0 (0.00%)	0 (0.00%)	0 (0.00%)	0 (0.00%)	0 (0.00%)	0 (0.00%)
Total	201 (68.14%)	4 (1.36%)	35 (11.86%)	84 (28.47%)	0 (0.00%)	196 (66.44%)	55 (18.64%)	67 (22.71%)	204 (69.15%)	149 (50.51%)	146 (49.49%)	9 (3.05%)	253 (85.76%)	226 (76.61%)	1 (0.34%)

Table S5. Drug resistance Pattern of *S.* Typhimurium ST34.

MDR	Pattern (93)	Number (%)
1	TE	4 (1.65%)
	NA	1 (0.41%)
	AMP	1 (0.41%)
	SUL	1 (0.41%)
2	NA+TE	1 (0.41%)
	NA+OFX+TE	1 (0.41%)
	S+TE	1 (0.41%)
	C+NA	1 (0.41%)
	AMP+SUL	4 (1.65%)
	SUL+TE	1 (0.41%)
	AMP+CTX+TE	1 (0.41%)

3	NA+FFC+TE	6 (2.47%)
	S+SUL+TE	9 (3.70%)
	C+FFC+SUL+TE	1 (0.41%)
	AMP+S+SUL	14 (5.76%)
	AMP+NA+SUL	2 (0.82%)
	AMP+S+CTX+SUL	2 (0.82%)
	AMP+SUL+TE	1 (0.41%)
	AMP+C+FFC+SUL	1 (0.41%)
4	NA+FFC+SUL+TE	2 (0.82%)
	C+NA+FFC+SUL+TE	1 (0.41%)
	AMP+CTX+NA+FFC+TE	1 (0.41%)
	S+FFC+SUL+TE	1 (0.41%)
	S+NA+SUL+TE	1 (0.41%)
	C+S+FFC+SUL+TE	8 (3.29%)
	AMP+FEP+S+CTX+SUL+TE	2 (0.82%)
	AMP+S+NA+SUL	2 (0.82%)
	AMP+C+NA+SUL	1 (0.41%)
	AMP+S+CTX+SUL+TE	1 (0.41%)
	AMP+S+SUL+TE	6 (2.47%)
	AMP+NA+SUL+TE	2 (0.82%)
	AMP+C+NA+FFC+SUL	1 (0.41%)
5	AMP+C+NA+CIP+FFC+SUL+TE	5 (2.06%)
	C+CN+NA+FFC+SUL+TE	4 (1.65%)
	AMP+C+NA+FFC+SUL+TE	9 (3.70%)
	AMP+C+CTX+NA+FFC+SUL+TE	1 (0.41%)
	CN+NA+FFC+SUL+TE	1 (0.41%)
	AMP+NA+FFC+SUL+TE	5 (2.06%)
	AMP+CTX+NA+FFC+SUL+TE	2 (0.82%)
	AMP+C+NA+CIP+OFX+FFC+SUL+TE	2 (0.82%)
	S+NA+OFX+FFC+SUL+TE	1 (0.41%)
	AMP+NA+OFX+FFC+SUL+TE	1 (0.41%)
	S+NA+FFC+SUL+TE	2 (0.82%)
	C+CN+NA+OFX+FFC+SUL+TE	1 (0.41%)
	AMP+S+NA+SUL+TE	1 (0.41%)
	S+CN+NA+FFC+TE	1 (0.41%)
	C+NA+CIP+OFX+PB+SUL+TE	1 (0.41%)
	C+S+CN+FFC+SUL+TE	1 (0.41%)
	C+CN+NA+CIP+OFX+FFC+SUL+TE	1 (0.41%)
	AMP+C+S+CTX+CN+FFC+SUL	1 (0.41%)
	ACSSuT+CTX+FFC	2 (0.82%)
	AMP+C+CTX+NA+CIP+OFX+FFC+SUL+TE	1 (0.41%)
6	AMP+S+CTX+NA+FFC+SUL+TE	1 (0.41%)
	AMP+C+CTX+NA+FFC+PB+SUL+TE	1 (0.41%)

	C+S+CN+NA+FFC+SUL+TE	4 (1.65%)
	AMP+C+CN+NA+CIP+FFC+SUL+TE	5 (2.06%)
	AMP+C+CTX+CN+NA+FFC+SUL+TE	1 (0.41%)
	AMP+CN+NA+FFC+SUL+TE	2 (0.82%)
	AMP+C+NA+CIP+FFC+PB+SUL+TE	1 (0.41%)
	ACSSuT+NA+CIP+FFC	4 (1.65%)
	AMP+C+CN+NA+FFC+SUL+TE	7 (2.88%)
	AMP+C+CN+NA+CIP+OFX+FFC+SUL+TE	12 (4.94%)
	AMP+C+CTX+NA+CIP+OFX+FFC+PB+SUL+TE	1 (0.41%)
	AMP+C+CTX+CN+NA+CIP+FFC+SUL+TE	1 (0.41%)
	ACSSuT+NA+FFC	6 (2.47%)
	AMP+S+NA+FFC+SUL+TE	6 (2.47%)
	AMP+C+CTX+CN+NA+CIP+OFX+FFC+SUL+TE	1 (0.41%)
	AMP+S+NA+OFX+FFC+SUL+TE	18 (7.41%)
	AMP+S+CTX+NA+CIP+OFX+FFC+SUL+TE	1 (0.41%)
	AMP+S+NA+CIP+OFX+FFC+SUL+TE	1 (0.41%)
	C+S+CN+NA+CIP+OFX+FFC+SUL+TE	1 (0.41%)
	AMP+C+CN+NA+OFX+FFC+SUL+TE	2 (0.82%)
	AMP+C+S+CN+NA+FFC+TE	1 (0.41%)
	ACSSuT+NA+OFX+FFC	1 (0.41%)
	AMP+C+S+CN+NA+FFC+SUL	1 (0.41%)
	AMP+C+S+CN+OFX+FFC+SUL	1 (0.41%)
	ACSSuT+AMP+C+S+CTX+CN+FFC	1 (0.41%)
	ACSSuT+CTX+NA+CIP+OFX+FFC	2 (0.82%)
	ACSSuT+NA+CIP+OFX+FFC	2 (0.82%)
	ACSSuT+CTX+NA+FFC	1 (0.41%)
	AMP+S+NA+FFC+PB+SUL+TE	1 (0.41%)
	AMP+S+CN+NA+FFC+SUL+TE	1 (0.41%)
	AMP+C+CN+NA+FFC+PB+SUL+TE	1 (0.41%)
	ACSSuT+CN+NA+FFC	10 (4.12%)
	AMP+C+CN+NA+CIP+FFC+PB+SUL+TE	1 (0.41%)
	ACSSuT+CTX+CN+NA+FFC	2 (0.82%)
7	ACSSuT+CN+NA+OFX+FFC	2 (0.82%)
	AMP+FEP+C+CTX+CN+IPM+NA+FFC+SUL+TE	1 (0.41%)
	ACSSuT+CTX+CN+NA+OFX+FFC	1 (0.41%)
	ACSSuT+CTX+CN+NA+CIP+FFC	1 (0.41%)
	ACSSuT+CN+NA+CIP+OFX+FFC	9 (3.70%)
	AMP+C+CTX+CN+NA+CIP+OFX+FFC+PB+SUL+TE	1 (0.41%)
	ACSSuT+CTX+NA+CIP+OFX+FFC+PB	1 (0.41%)

Table S6. Drug resistance Pattern of *Salmonella* Typhimurium ST19.

MDR	Pattern (22)	Number (%)
0	-	8 (17.02%)
1	NAL	4 (8.51%)

	SUL	1 (2.13%)
	FFC	1 (2.13%)
	TE	4 (8.51%)
2	C+SUL	1 (2.13%)
	AMP+FEP+CTX+SUL	1 (2.13%)
	C+FFC+SUL	1 (2.13%)
	NAL+SUL	3 (6.38%)
3	AMP+S+NAL+SUL	1 (2.13%)
	AMP+C+FFC+SUL	3 (6.38%)
	C+FFC+SUL+TE	4 (8.51%)
	OFX+SUL+TE	1 (2.13%)
4	AMP+C+S+FFC+SUL	5 (10.64%)
	S+NAL+SUL+TE	1 (2.13%)
	AMP+C+FFC+SUL+TE	1 (2.13%)
	C+NAL+FFC+SUL+TE	1 (2.13%)
5	S+CN+NAL+FFC+SUL+TE	1 (2.13%)
	AMP+C+S+CN+FFC+SUL	1 (2.13%)
	AMP+S+NAL+SUL+TE	2 (4.26%)
	C+CN+NAL+FFC+SUL+TE	1 (2.13%)
7	ACSSuT+CN+NAL+FFC	1 (2.13%)
